# Chloroform as an Anæsthetic

**Published:** 1889-12

**Authors:** 


### Chloroform as an Anæsthetic.

A contributor to *The Lancet* states that in the medical journals for the last ten years there are reported one hundred and twenty (if not more) cases of death under chloroform. Many of these are very imperfectly described, but in at least forty-nine cases the patients were in good general health at the time of administration, and required an anæsthetic merely for the performance of some minor operation; e. g., extraction of teeth (eleven cases of death), reduction of dislocations (nine cases), eye operations, fistulæ, and so on. In some fifty-nine cases death occurred before the commencement of the operation, and so was clearly due to the chloroform alone. In about twenty of the cases it is noted that chloroform had been successfully given on previous occasions, in one as many as eight different times before the fatal administration. It is evident from the foregoing that chloroform is uncertain in its action; that not only do people die while under chloroform, but also from it; frequently, too, even when it is used by skillful hands. Of course, it is possible to retort that "it was not properly given," which may be correct. This will not alter the fact that these accidents prove chloroform to be a powerful agent, very difficult to administer properly; indeed, so difficult and dangerous that it is scarcely suitable for a routine anæsthetic, when a drug less powerful for evil can replace it.

The nauseous flavor and the sense of suffocation from ether can be entirely done away with by the use of nitrous oxide, and its inhalation made more agreeable than even that of chloroform, while the patient quickly becomes unconscious without the struggling so common with chloroform. The writer goes on to say, "I have not yet found a single patient who has once inhaled ether preceded by nitrous oxide complain of suffocation, or object to take it again on the ground of its unpleasantness.

"The readiness with which chloroform effects the heart, the smallness of a fatal dose, and especially the ease and suddenness with which such a dose can be inhaled, almost by a couple of deep inspirations, will make its safe exhibition always a difficult task to invariably accomplish. Having had many years' experience, I have gradually come to believe chloroform to be a less safe anæsthetic than ether."

